# NMR Study on the Interaction of Trehalose with Lactose and Its Effect on the Hydrogen Bond Interaction in Lactose

**DOI:** 10.3390/molecules18089735

**Published:** 2013-08-14

**Authors:** Eric Morssing Vilén, Corine Sandström

**Affiliations:** Department of Chemistry, Swedish University of Agricultural Sciences, Biocenter P.O. Box 7015, Uppsala SE-75007, Sweden; E-Mail: corine.sandstrom@slu.se

**Keywords:** trehalose, lactose, sucrose, NMR, hydroxy protons, hydrogen bonding, interaction

## Abstract

Trehalose, a well-known stress-protector of biomolecules, has been investigated for its effect on the mobility, hydration and hydrogen bond interaction of lactose using diffusion-ordered NMR spectroscopy and NMR of hydroxy protons. In ternary mixtures of trehalose, lactose and water, the two sugars have the same rate of diffusion. The chemical shifts, temperature coefficients, vicinal coupling constants and ROE of the hydroxy protons in trehalose, lactose and sucrose were measured for the disaccharides alone in water/acetone-*d_6_* solutions as well as in mixtures. The data indicated that addition of trehalose did not change significantly the strength of the hydrogen bond interaction between GlcOH3 and GalO5' in lactose. Small upfield shifts were however measured for all hydroxy protons when the sugar concentration was increased. The chemical shift of the GlcOH3 signal in lactose showed less change, attributed to the spatial proximity to GalO5'. Chemical exchange between hydroxy protons of lactose and trehalose was observed in the ROESY NMR spectra. Similar effects were observed with sucrose indicating no specific effect of trehalose at the concentrations investigated (73 to 763 mg/mL) and suggesting that it is the concentration of hydroxy groups more than the type of sugars which is guiding intermolecular interactions.

## 1. Introduction

**Scheme 1:**
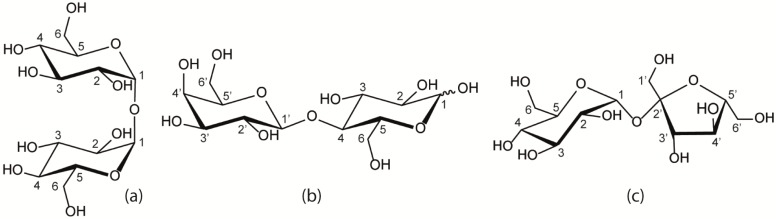
Schematic representation of (**a**) trehalose (**b**) lactose and (**c**) sucrose.

Trehalose and sucrose are non-reducing, naturally occurring disaccharides with the same molecular formula (C_12_H_22_O_11_). They are constituted of α-d-glucose-(1→1)-α-d-glucose and of α-d-glucose-(1→2)-β-d-fructose, respectively (Scheme 1). Trehalose and sucrose are well known for their ability to protect various biomolecules such as proteins and membranes from different kinds of stress, including freezing, heating, desiccation and osmotic shock [1,2,3,4,5]. The detailed molecular mechanism underlying their bioprotecting abilities and in particular the superior efficiency of trehalose is however still a matter of debate and under investigation. Several hypotheses have been proposed to explain the effect of trehalose at the molecular level on the structure of biomolecules: (1) the water replacement hypothesis in which trehalose binds through hydrogen bonds to the biomolecule instead of water [6]; (2) the preferential hydration hypothesis or water entrapment hypothesis [7]; (3) the high viscosity hypothesis in which viscosity effects cause motional inhibition [8,9] and (4) the water destructuring effect mechanism where trehalose effectively disturbs the structure of water needed for proper ice formation [10,11,12,13]. These different mechanisms are not necessarily mutually exclusive and have found support in different experimental and theoretical investigations.

The property most often implicated in the stabilizing effect of sugars is their interaction with water. Both experiments and simulations show a remarkable slowing down of the water mobility in sugar solution, often explained as being due to the formation of sugar-water hydrogen bonds [14,15,16,17,18,19,20]. The overall water dynamical retardation is found to strongly increase with the sugar concentration and the number of water-sugar hydrogen bonds to decrease with increasing solute concentration when sugar-sugar contacts become important. Trehalose has been assigned a slightly stronger solvation leading to a stronger interaction with water. Molecular dynamics (MD) simulations have shown that in the 0%–66% (w/w) concentration range trehalose slow down water dynamics [14], with the effects increasing considerably at concentrations above 40% (w/w).

The ability of carbohydrates to stabilize proteins in aqueous solution has been attributed to the preferential hydration of protein that occurs when it is dissolved in a sugar solution. Experimental and simulation results suggest that sugars are preferentially excluded from the protein surface. Therefore their bioprotective effect is thought to be partially due to the significant slowing down they induce on the dynamics of protein hydration molecules. The disaccharides trehalose, maltose and sucrose have been shown to decrease the flexibility of lysozyme [21]. Trehalose exhibited superior protecting capabilities partially due to its higher hydration number and higher ability to slow down water dynamics. A recent study by Lupi *et al.* [22] suggests however that the concentration of hydroxyl groups is the relevant parameter, guiding both water-sugar and sugar-sugar interactions, and no specific effect of trehalose was detected that could provide an explanation for the better bioprotective features of this disaccharide.

Thus, despite number of studies using different techniques like computer modeling [12,13,14,16,19,20,21,22,23,24,25,26,27,28,29,30,31,32,33], light scattering [3,6,7,10,11,13,14,15,17,22,34,35,36], neutron scattering [37,38,39,40,41], neutron diffraction [42] and NMR [32,43,44,45,46,47], there is yet no definitive answer on the superior stabilizing capacity of trehalose or its solvent perturbation capabilities and if that can be attributed to its hydration.

Since the bioprotective properties of trehalose and sucrose are thought to be associated to interaction with water and involve their hydroxyl groups, one way of investigating such interactions is to study the hydroxy protons by NMR spectroscopy. Lactose is a reducing disaccharide constituted of β-d-galactose 1→4 linked to d-glucose. It has been shown that the structure of lactose in aqueous solution is characterized by a transient hydrogen bond interaction between OH3 of glucose (GlcOH3) and O5' of galactose (GalO5') [48]. The goal of the present work was to determine if and how the hydration and the strength of the hydrogen bond is affected by the addition of trehalose and sucrose. For this, we have studied the hydroxy protons of the sugars in the different binary and ternary sugar-water solutions. The chemical shifts (δ), chemical shift differences (Δδ), coupling constants, (^3^*J*_CH, OH_), temperature coefficients (dδ/dΤ) and rotating frame nuclear Overhauser effects (ROE) of the hydroxy protons of lactose alone in aqueous solution and in the presence of trehalose or sucrose were measured over a range of concentrations. The diffusion of lactose, water and trehalose in binary and ternary systems was also studied to determine whether lactose has the same mobility as trehalose.

## 2. Results and Discussion

### 2.1. Diffusion in Binary and Ternary Systems

The diffusion properties of trehalose, sucrose and lactose in aqueous solutions have been widely investigated using different types of experimental and theoretical methods [34,44,46,49,50,51]. Thus, in this work, DOSY experiments were used to qualitatively compare the self-diffusion of trehalose, lactose and water at different concentrations and temperatures. No separation between the α- and β-forms of lactose was achieved in the DOSY spectra.

#### 2.1.1. Binary Water/Trehalose and Water/Lactose Systems

**Figure 1:**
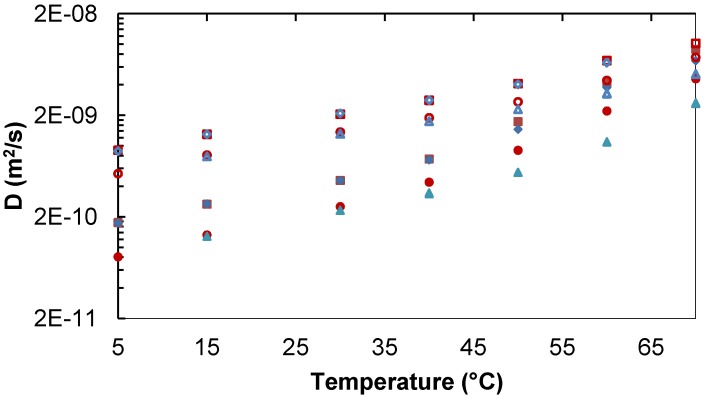
Diffusion of trehalose, lactose and water in trehalose/water and lactose/water solutions. Lac 34 mg/mL: 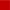
Lac, 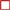
D_2_O. Lac 253 mg/mL: 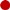
Lac, 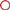
D_2_O. Tre 34 mg/mL: 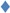
Tre, 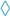
D_2_O. Tre 258 mg/mL: 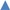
Tre, 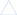
D_2_O.

At low sugar concentration and below 40 °C, trehalose and lactose have similar diffusion while at higher temperature, lactose diffusion is slightly faster (Figure 1). Water has the same diffusion in both systems and over the entire range of temperatures. For higher sugar concentrations (>253 mg/mL), the diffusion of the two disaccharides is similar only at the lowest temperatures. At temperatures above 30 °C trehalose shows a slower diffusion than lactose, the difference increasing with the temperature (Figure 1). The diffusion of water is slower in presence of trehalose at temperatures above 45 °C, as also shown before [44,46]. The ratio between the water diffusion and the sugar diffusion increases when the sugar concentrations increase and it decreases with increasing temperature. These results are in agreement with previous studies which have shown that higher concentration of disaccharides result in slower diffusion rates and that higher temperature give faster diffusion rates [34,44,46,50]. Trehalose has also been shown to have slower diffusion than a number of disaccharides other than lactose [44,46,51].

#### 2.1.2. Ternary Water/Trehalose/Lactose Systems

**Figure 2:**
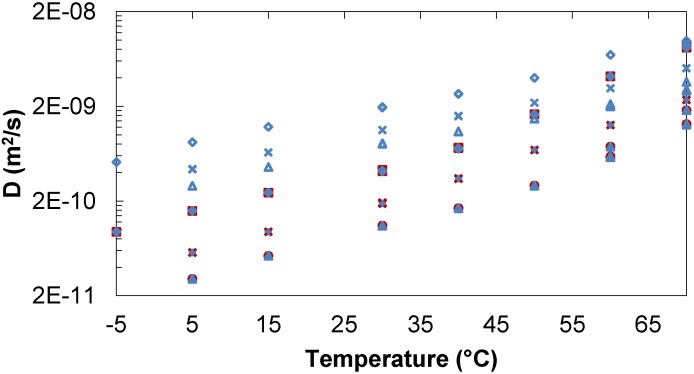
Diffusion of trehalose, lactose and water in trehalose/lactose/water solutions. Tre/Lac 34/34 mg/mL: 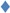 Tre, 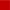
Lac, 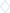
D_2_O. Tre/Lac 170/170 mg/mL: 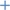
Tre, 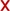
Lac, 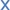
D_2_O. Tre/Lac 254/254 mg/mL: 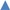
Tre, 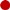
Lac, 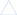
D_2_O.

When in mixtures, trehalose and lactose have similar diffusion for a given temperature and concentration over the entire range of temperatures (−5 to +70 °C) and concentrations (34/34–254/254 mg/mL) investigated. Water has a faster diffusion rate than the sugars but the difference decreases as the temperature increases (Figure 2).

#### 2.1.3. Comparison of Trehalose/Lactose/Water and Trehalose/Glucose/Water systems

**Figure 3:**
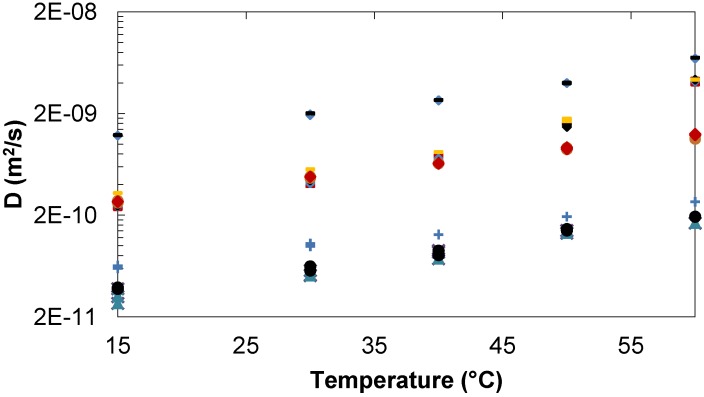
Diffusion of trehalose, lactose, glucose and water in trehalose/lactose/water and trehalose/glucose/water solutions. Tre/Lac 34/34 mg/mL: 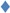
Tre, 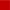
Lac, 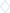
D_2_O. Tre/Glc 35/20 mg/mL: ♦Tre, 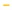
Glc, 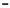
D_2_O. Tre/Lac 780/63 mg/mL: 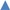
Tre, 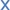
Lac, 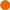
D_2_O. Tre/Glc 785/67 mg/mL: ●Tre, 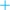
Glc, 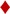
D_2_O.

The diffusion of water is similar in the trehalose/lactose and trehalose/glucose systems at 34/34 mg/mL and 35/20 mg/mL. While trehalose and lactose have the same diffusion over the entire temperature range glucose showed, as expected because of its smaller size, a faster diffusion rate between 10 and 50 °C. At higher temperature, 70 °C, the diffusion of glucose, trehalose and water is converging. When the concentration of trehalose is in excess compared to that of lactose (780 *versus* 63 mg/mL, Figure 3) similar diffusion is observed over the entire temperature range. Glucose, in solution with large excess of trehalose, has a faster diffusion over the whole temperature range (Figure 3). The difference between trehalose and glucose diffusion is slightly larger in the 35/20 mg/mL system than in the 785/67 mg/mL mixture and that might be due to an increased number of sugar-sugar interactions at the highest concentrations [31,43].

Sugars have been shown to have a strong tendency to self-association in aqueous solution, leading to concentration dependent clustering, affecting the solution dynamic properties such as diffusion. The differences become measurable only at concentrations where direct contact between the sugar molecules becomes probable, that is concentration of approximately 50% (w/w). Thus, Rampp *et al*. [46] have shown that up to a concentration of circa 30% (w/w), the viscosity and self-diffusion coefficients of the disaccharides, sucrose, trehalose, leucrose and allosucrose are nearly the same for a given temperature and concentration. At higher concentrations, the viscosities in the trehalose solutions was higher and the self-diffusion of trehalose and water slower than in the three other mixtures. Pulsed-gradient spin-echo NMR and MD simulations have shown [44] that the diffusion of sucrose and trehalose are comparable at low sugar concentrations but differ from each other with increasing concentrations. The higher mobility of sucrose was attributed to its small hydration number and more compact shape. At concentrations below 72% (w/w), the diffusion of water was independent of the type of sugar while in 80% (w/w) disaccharide solutions water was diffusing twice as fast in sucrose solutions as in trehalose solutions. Sapir *et al*. [31] have studied the self-association of trehalose in aqueous solutions over a wide range of concentrations using MD simulations complemented by vapor pressure osmometry experiments and have shown that trehalose molecules aggregate into clusters that increase in size with increasing concentration. At the percolation threshold, estimated in simulations to be at molalities between 1.5 and 2.2 m, infinite clusters form, and the concentration of finite cluster drops. Simulations by Lerbret *et al*. [12] have also shown that trehalose molecules form clusters that increase in size with an increase in concentration. Also Winther *et al*. [43] concluded using NMR spin relaxation rate measurements of ^2^H and ^17^O, in trehalose and water respectively, that trehalose clustering occur, although to a limited extent, at the low concentration of 0.1 M. Thus at the concentrations investigated in the present work (≤50% (w/w)), the formation of clusters is probably not pronounced.

### 2.2. NMR of Hydroxy Protons

According to the DOSY experiments, the diffusion of trehalose and lactose, when in mixture, are comparable at the temperatures at which the hydroxy protons will be studied. The occurrence of hydrogen bond interactions in aqueous solutions of trehalose, lactose and sucrose have been previously investigated using MD simulation [23,28,30,52] as well as NMR of hydroxy protons [48,53,54,55]. Thus, hydroxy protons involved in hydrogen bonding are expected to have smaller temperature coefficients, a slower rate of exchange with water, and coupling constants which do not represent conformational averaging but instead indicate a restricted rotation around the H–C–O–H bond. In supercooled lactose, the presence of a hydrogen bond between GalO5' and GlcOH3 was deduced [48] from the decrease in the chemical exchange rate of GlcOH3 as well as from the small value of its ^3^*J*_CH, OH_ (3.1 Hz) that deviate from the value of 5.5 ± 0.5 Hz which would indicate free rotation around the H–C–O–H bond [56]. In sucrose the existence of a transient hydrogen bond between the GlcOH2 and FruOH1' groups was evidenced under supercooled conditions from the chemical exchange cross-peak observed in the ROESY spectra between glucosyl OH2 and fructosyl OH1 protons [55]. For trehalose, the ^3^*J*_CH, OH_ and ^2,3^*J*_C,OH_ of the hydroxy protons, measured at a concentration of 1.7 M in a 97:3 D_2_O/H_2_O solvent at −3 °C, suggested that intramolecular hydrogen bonding interaction was not present [53] in good agreement with MD simulation and X-ray data [25,57,58].

In the present work, the NMR experiments were performed using a 90% H_2_O/10% acetone-*d_6_* solvent system. The chemical shifts, δ, chemical shift differences, Δδ, temperature coefficients, dδ/dT, and vicinal coupling constants, ^3^*J*_CH, OH_, for trehalose, lactose and sucrose at different concentrations, alone in solutions or in mixtures of disaccharides are listed in Table 1, Table 2, Table 3 and Table 4.

#### 2.2.1. Binary Systems

**Figure 4:**
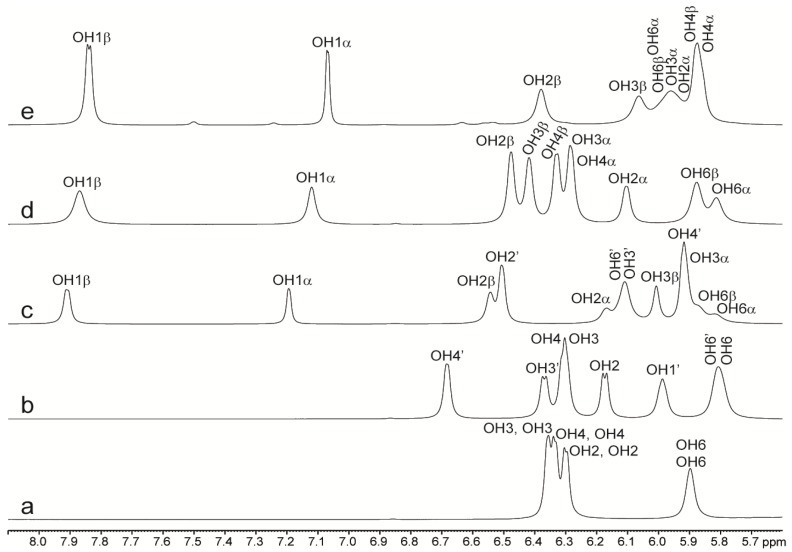
(**a**) ^1^H NMR spectra of the hydroxy proton region of (**a**) trehalose, (**b**) sucrose, (**c**) lactose, (**d**) glucose and (**e**) galactose at −5 °C in 90% H_2_O/10% Acetone-*d_6_*. Notice the large upfield shifts of GlcOH3(α/β) in spectrum c compared to spectrum **d**.

The NMR data for the disaccharides alone in solution (Table 1) are in good agreement with what has been reported in the previous studies described above, but performed under slightly different experimental conditions [48,53,55]. Besides temperature coefficients, coupling constants and chemical exchange, it has been shown previously that the chemical shift difference Δδ (chemical shift of a hydroxy proton in an oligosaccharide minus chemical shift of the hydroxy proton in the constituent monosaccharide) can also be used as a conformational probe to study hydrogen bond interaction, hydration and spatial proximity to other hydroxy protons, ring oxygen or bulky substituents [59,60,61]. Thus a positive Δδ indicate that the hydroxy proton in an oligosaccharide is downfield shifted if compared to the shift in the monosaccharide and reflect spatial proximity to another hydroxy proton. A negative Δδ indicate spatial proximity to non-protonated O5 oxygen or to a bulky substituent and is attributed to a reduced hydration of the hydroxy proton in the oligosaccharide if compared to the hydration in the building block monosaccharide. In lactose all the hydroxy protons have small Δδ <│0.2│ ppm with the exception of the GlcOH3 α- and β-anomeric signals (α/β) which are upfield shifted by 0.4 ppm (Δδ) if compared to the chemical shift in the monosaccharide (Figure 4).

Such an upfield shift was also observed by Poppe *et al*. [48] who pointed out that hydroxy protons involved in hydrogen bonds should be deshielded. In agreement with previous studies [59] this large negative Δδ indicates spatial proximity to GalO5'. In trehalose, OH2 has a positive Δδ of 0.21 ppm suggesting as mentioned above spatial proximity to another hydroxy group. Due to symmetry, only one set of NMR signals is observed for the two glucose residues. Data from literature [19,25,26,29] show that the OH2 groups are not close to each other (~5 Å) or to any other non-vicinal OH group with the exception of OH6 in some computer modeled low energy states. Some of these studies have shown residence times for water molecules around the OH2 groups and O1 that are longer than for any of the other hydroxyl groups [19,26]. Nunes *et al*. [29] found in the gas phase one high energy conformer, where the two OH2 groups are very close (~2.5Å) and show potential hydrogen bonding. Conformers exhibiting potential hydrogen bonding between OH2 and OH6' or O5' and with an increased relative population in solution compared to the gas phase were however also found [29]. Other simulation studies [23,28] also reveal transient, direct or water mediated, OH2–OH6' inter-residue hydrogen bonds in trehalose. Consequently the positive Δδ of OH2 of 0.21 ppm might reflect transient direct or water bridged interaction to the OH6' group or the existence of water bridged interaction over the glycosidic linkage between the two OH2 groups. None of the hydroxy protons in sucrose exhibited Δδ values larger than │0.06│ ppm.

**Table 1:** ^3^*J*_CH, OH_ and dδ/dT values for lactose and trehalose OH-protons in disaccharide/water binary mixtures. All coupling constants could not be extracted due to broad signals and/or overlap.

**Lactose**	**35 mg/mL**		**203 mg/mL**	
	**^3^*J*_CH, OH_/dδ/dT**		**^3^*J*_CH, OH_/dδ/dT**	
GlcOH1α	4.2/−8.7		4.4/−8.4	
GlcOH1β	5.5/−9.0		6.5/−9.8	
GlcOH2α	5.9/−11.4		----/−11.3	
GlcOH2β	4.9/−11.8		4.8/−11.6	
GlcOH3α	3.5/−10.4		----/−10.2	
GlcOH3β	3.5/−10.8		3.3/−10.8	
GlcOH6α	----/−11.1		----/−10.7	
GlcOH6β	----/−11.7		----/−10.9	
GalOH2	5.4/−10.7		5.4/−10.6	
GalOH3	5.9/−10.5		----/−10.3	
GalOH4	5.1/−10.9		----/−10.8	
GalOH6	5.6/−12.7		----/−12.3	
**Trehalose**	**35 mg/mL**	**206 mg/mL**		**338 mg/mL**
	**^3^*J*_CH, OH_/dδ/dT**	**^3^*J*_CH, OH_/dδ/dT**		**^3^*J*_CH, OH_/dδ/dT**
OH2	6.9/−11.0	7.0/−10.8		7.0/−10.6
OH3	5.2/−11.8	5.1/−11.6		5.0/−11.3
OH4	6.5/−10.5	6.5/−10.4		6.4/−10.1
OH6	----/−11.8	----/−11.4		----/−10.9
OH6	----/−11.8	----/−11.4		----/−10.9

**Figure 5:**
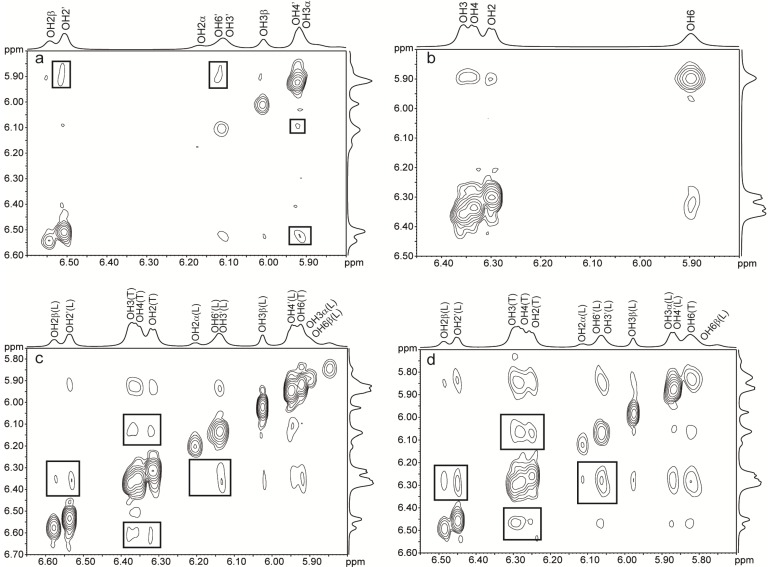
ROESY ^1^H-NMR spectra at –5 °C of (**a**) lactose 203 mg/mL, (**b**) trehalose 206 mg/mL, (**c**) trehalose/lactose 37/36 mg/mL mixture, mixing time 80 ms, (**d**) trehalose/lactose mixture 256/256 mg/mL, mixing time 100 ms.

Through space interactions can be detected by NOESY experiments, but discrimination between signals from chemical exchange and from dipolar relaxation can only be achieved via ROESY experiments. Thus, ROE cross-peaks originating from chemical exchange have the same sign as the diagonal peaks, while cross-peaks originating from dipolar relaxation have the opposite sign. The 2D ROESY NMR spectra of trehalose showed chemical exchange between OH3/4 and OH6 as well as between OH2 and OH6 (Figure 5b). Since there is only one set of NMR signals for the two glucose residues, one cannot distinguish between intra- or inter-residual chemical exchange. According to simulations [23,28,31] OH3/4–OH6 exchange occur intra- as well as inter-residually while OH2–OH6 exchange occurs inter-residually. The dominant exchange process is occurring between hydroxy protons and water, but upon increasing the concentration of trehalose from 35 to 206 mg/mL the exchange between hydroxy protons increased as compared to the exchange between water and trehalose (data not shown). In lactose, only weak cross-peaks between the hydroxy protons of galactose were observed (Figure 5a). The GlcOH3–GalO5' interaction in lactose cannot be detected with ROESY since it is not possible to observe hydrogen bonding to ring oxygens.

**Figure 6:**
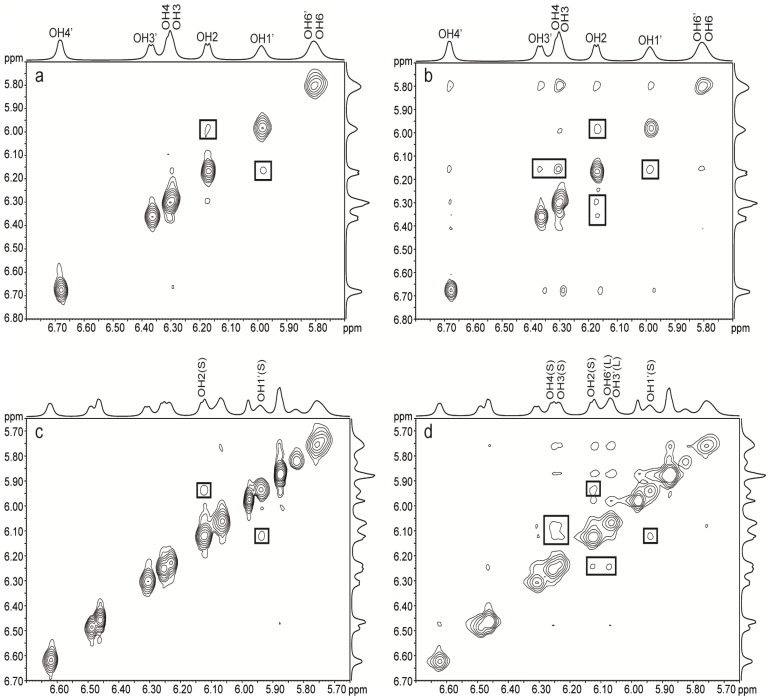
ROESY ^1^H-NMR spectra at –5 °C of (**a**) sucrose 202 mg/mL, mixing time 40 ms, (**b**) sucrose 202 mg/mL, mixing time 80 ms, (**c**) sucrose/lactose 178/181 mg/mL mixture, mixing time 40 ms, (**d**) sucrose/lactose mixture 178/181 mg/mL, mixing time 80 ms.

In sucrose the previously reported chemical exchange between GlcOH2 and FruOH1' [54,55] was observed (Figure 6a) as well as a weak exchange between GlcOH2 and FruOH3' (Figure 6b). The observation of this additional cross-peak in the ROESY spectra might be due to the higher sugar concentration used in our study. The intramolecular hydrogen bonds in sucrose might be concentration dependent and the existence of hydrogen bonds FruOH1'–GlcO2, FruOH3'–GlcO2 and FruOH6'–GlcO5 at higher concentrations has been postulated based on ^13^C isotope effects [62]. The existence of the first two hydrogen bonds at high concentration in solution implies that there are two conformers that are in fast interconversion due to flexibility around the glycosidic linkage in solution. In the crystal state, only FruOH1'–GlcO2 and FruOH6'–GlcO5 are observed [63]. As for lactose, the GlcO5–FruOH6' hydrogen bond cannot be detected with ROESY. Furthermore no information on the donor in hydrogen bond interactions is obtained with ROESY. The interactions between FruOH1'–GlcOH2 and FruOH3'–GlcOH2 together with the exchange cross-peaks between GlcOH3/OH4 and GlcOH2 (Figure 6b) are in line with results from simulations performed by Engelsen *et al*. [26] showing longer residence times for water molecules along one side of the sucrose molecule. Longer residence times could mean that the hydroxy protons are more likely to exchange between sugar hydroxy groups through water molecules that stay close to the sugar for longer times before being replaced by bulk water molecules. When comparing data from MD simulations and NMR experiments it should however be noted that MD provides information on dynamic events ranging from circa the nanosecond down to the sub-femtosecond scale, while NMR under normal conditions use perturbation times in the micro and millisecond range and thereby only provide time averaged dynamic information about sub-microsecond events.

#### 2.2.2. Ternary Systems

For the lactose/trehalose/water mixture, Table 2 and Table 3 show that increasing the concentration of sugars results in a decrease by ≤ 1 ppb/°C in temperature coefficients.

**Table 2:** ^3^*J*_CH, OH_, Δδ_mix_ and dδ/dT values for trehalose OH-protons in trehalose/lactose/water mixtures. All coupling constants could not be extracted due to broad signals and/or overlap.

Tre/Lac	37 + 36 mg/mL	256 + 256 mg/mL	383 + 380 mg/mL	593 + 54 mg/mL
Trehalose	^3^*J*_CH, OH_/Δδ_mix_/dδ/dT	^3^*J*_CH, OH_/Δδ_mix_/dδ/dT	^3^*J*_CH, OH_/Δδ_mix_/dδ/dT	^3^*J*_CH, OH_/Δδ_mix_/dδ/dT
OH2	7.0/−0.005/−11.0	6.8/−0.066/−10.3	----/−0.111/−10.4	6.7/−0.092/−10.4
OH3	5.2/−0.006/−11.8	4.6/−0.077/−11.0	----/−0.132/−10.9	4.7/−0.106/−11.0
OH4	6.6/−0.007/−10.4	6.4/−0.076/−9.8	----/−0.125/−10.0	6.3/−0.104/−10.0
OH6	----/−0.011/−11.8	----/−0.104/−10.5	----/−0.167/−10.3	----/−0.144/−10.2
OH6	----/−0.011/−11.8	----/−0.104/−10.5	----/−0.167/−10.3	----/−0.144/−10.2

Δδ_mix_: δ(Trehalose/Lactose mg/mL) – δ(Trehalose/Lactose 37 + 36 mg/mL) , except for the 37 + 36 mg/mL mixture where Δδ_mix_: δ (Trehalose/Lactose 37 + 36 mg/mL) – δ(Lactose 35 mg/mL).

This is similar to what is observed upon increasing the sugar concentrations in the binary systems (Table 1). The temperature dependence of exchangeable protons is commonly used as hydrogen bond indicators and low temperature coefficients (<│3│ ppb/°C) have been associated to strong hydrogen bonds. All hydroxy protons, except the anomeric hydroxy protons in lactose have temperature coefficient (dδ/dT) values > │9.5│ ppb/°C, indicating that they are not involved in strong intermolecular hydrogen bond interactions. There are no significant changes in the values of coupling constants with the exception of ^3^*J*_CH, OH_ of GlcOH3β which is decreasing from ~3.4 Hz to ~2.8 Hz when the concentration of sugars increase from 73 to 512 mg/mL (Table 3).

**Table 3:** ^3^*J*_CH, OH_, Δδ_mix_ and dδ/dT values for lactose OH-protons in trehalose/lactose/water mixtures. All coupling constants could not be extracted due to broad signals and/or overlap.

Tre/Lac	37 + 36 mg/ml	256 + 256 mg/ml	383 + 380 mg/ml	593 + 54 mg/ml
Lactose	^3^*J*_CH, OH_/Δδ_mix_/dδ/dT	^3^*J*_CH, OH_/Δδ_mix_/dδ/dT	^3^*J*_CH, OH_/Δδ_mix_/dδ/dT	^3^*J*_CH, OH_/Δδ_mix_/dδ/dT
GlcOH1α	4.1/−0.014/−8.6	3.7/−0.129/−7.6	----/−0.204/−7.4	----/−0.170/−7.8
GlcOH1β	6.4/−0.012/−9.1	5.9/−0.109/−8.1	----/−0.175/−8.6	5.9/−0.145/−9.0
GlcOH2α	6.1/−0.011/−11.3	5.7/−0.090/−10.5	----/−0.152/−10.5	5.7/−0.128/−10.4
GlcOH2β	4.9/−0.011/−11.8	4.6/−0.093/10.9	----/−0.155/−10.9	4.3/−0.128/−10.9
GlcOH3α	----/−0.003/−10.5	----/−0.043/−10.2	----/−0.072/−9.2	----/−0.060/−9.9
GlcOH3β	3.4/−0.005/−10.8	2.8/−0.049/−9.9	----/−0.088/−9.6	----/−0.072/−9.9
GlcOH6α	----/−0.011/−11.0	----/−0.097/−9.9	----/−0.156/−9.7	----/−0.133/−9.8
GlcOH6β	----/−0.011/−11.2	----/−0.091/−10.0	----/−0.144/−10.1	----/−0.125/−10.2
GalOH2	5.4/−0.009/−10.6	5.1/−0.088/−9.9	----/−0.145/−10.0	4.8/−0.117/−10.1
GalOH3	5.8/−0.008/−10.5	----/−0.075/−9.7	----/−0.128/−9.4	----/−0.101/−9.5
GalOH4	----/−0.008/−10.9	----/−0.083/−10.1	----/−0.138/−10.0	----/−0.112/−10.1
GalOH6	----/−0.011/−12.4	----/−0.089/−11.3	----/−0.140/−11.4	----/−0.118/−11.2

Δδ_mix_: δ(Trehalose/Lactose mg/mL) – δ(Trehalose/Lactose 37 + 36 mg/mL), except for the 37 + 36 mg/mL mixture where Δδ_mix_: δ(Trehalose/Lactose 37+36 mg/mL) – δ(Lactose 35 mg/mL).

**Figure 7:**
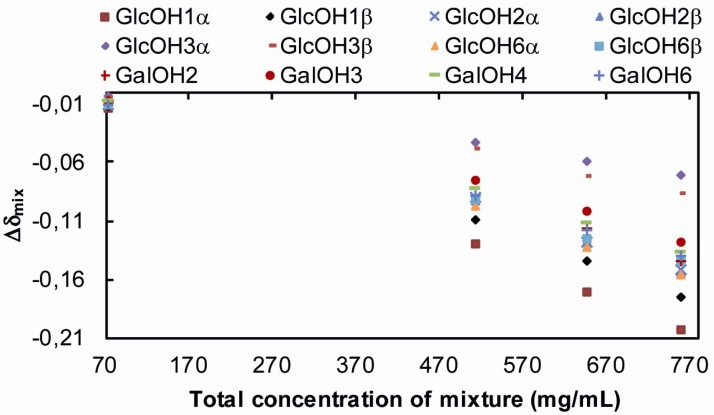
Δδ_mix_ values for the hydroxy protons of lactose in trehalose/lactose/water solutions as a function of the total sugar concentration.

Because of spectral overlap between GlcOH3(α) and GalOH4' of lactose, ^3^*J*_CH, OH_ of GlcOH3(α) could not be extracted. Small upfield shifts (Δδ_mix_, Table 3 and Figure 7) are measured for the hydroxy protons of lactose upon the addition of trehalose. The Δδ_mix_ increases as the sugar concentration increase. Similar upfield shifts are also measured for the hydroxy protons in trehalose. With the exception of the anomeric hydroxy protons the Δδ_mix_ is largest for the OH6 of both trehalose and lactose (Table 2, Table 3 and Figure 7). GlcOH3 in both α- and β-anomers experience a smaller upfield shift (Δδ_mix_ ~0.05 ppm) than the other secondary hydroxy protons (average Δδ_mix_ ~0.10 ppm) when comparing for example the 37/36 mg/mL and 256/256 mg/mL trehalose/lactose solutions (Table 3, Figure 7). For the 383/383 mg/mL mixture Δδ_mix_ is 0.08 for GlcOH3 and >0.14 ppm for the other hydroxy protons. The smaller Δδ_mix_ of GlcOH3α/β can be attributed to the spatial proximity of GlcOH3α/β to GalO5'.

ROESY spectra were recorded at different mixing times and different concentrations of lactose and trehalose. Besides the chemical exchange observed between hydroxy protons in trehalose and in lactose, chemical exchange between hydroxy protons of trehalose and lactose was also observed in the ternary systems (Figure 5c and d). The predominant exchange occurs between OH2, 3 and 4 of trehalose and GalOH2', 3', 4' and 6' of lactose but a cross-peak is also observed between GlcOH2 in lactose and OH3, 4 in trehalose. The cross-peaks are stronger between lactose and trehalose than between lactose or trehalose but direct comparison of cross-peak intensities is difficult due to signal overlap. The exchange between lactose and water hydroxy protons decreases slightly upon addition of trehalose, as well as the exchange between trehalose and water hydroxy protons. There is less chemical exchange between GlcOH3 of lactose and the hydroxy protons of trehalose. This lack of intermolecular interaction can be explained by the spatial proximity to GalO5', which shield the GlcOH3 group from interaction with the surroundings. Only ROEs originating from chemical exchange between hydroxy protons are detected and no ROEs due to dipolar relaxation between hydroxy and aliphatic protons or between aliphatic protons of trehalose and lactose are observed. The absence of dipolar interaction might be due to the fact that the chemical exchange observed between hydroxy protons of lactose and trehalose is water-mediated.

The effect of addition of sucrose and of glucose on the temperature coefficients and chemical shifts of the hydroxy protons of lactose are shown in Table 4. The results are very similar to what was obtained with trehalose. Upfield shifts comparable to what was measured for GlcOH3α/β and the other hydroxy protons of lactose in the trehalose/lactose mixtures were also observed for ternary mixtures of sucrose/lactose and glucose/lactose (Table 4). Upon addition of sucrose, a decrease in the value of ^3^*J*_CH, OH_ of GlcOH3β was observed, as in the trehalose/lactose mixture.

**Table 4:** ^3^*J*_CH, OH_, Δδ_mix_ and dδ/dT values for lactose OH-protons in sucrose/lactose/water and glucose/lactose/water mixtures. All coupling constants could not be extracted due to broad signals and/or overlap.

	Sucrose/Lactose	Glucose/Lactose
Lactose	178 + 181 mg/mL	239 + 240 mg/mL
	^3^*J*_CH, OH_/Δδ_mix_/dδ/dT	^3^*J*_CH, OH_/Δδ_mix_/dδ/dT
GlcOH1α	4.1/−0.112/−7.9	4.2/−0.137/−8.0
GlcOH1β	6.3/−0.096/−8.5	6.3/−0.120/−8.7
GlcOH2α	----/−0.090/−11.5	----/−0.091/−10.8
GlcOH2β	4.7/−0.091/−11.4	4.0/−0.095/−11.3
GlcOH3α	----/−0.045/−10.2	----/−0.038/−10.2
GlcOH3β	2.7/−0.050/−10.2	----/−0.053/−10.2
GlcOH6α	----/−0.086/−10.4	----/−0.100/−10.3
GlcOH6β	----/−0.078/−10.5	----/−0.098/−10.5
GalOH2	5.2/−0.077/−10.3	5.1/−0.090/−10.3
GalOH3	5.4/−0.075/−9.9	----/−0.075/−10.0
GalOH4	----/−0.077/−10.4	----/−0.087/−10.4
GalOH6	----/−0.071/−11.6	----/−0.095/−11.6

Δδ_mix_: δ (Sugar /Lactose mg/mL) – δ (Trehalose/Lactose 37 + 36 mg/mL).

Only weak chemical exchanges are observed in the ROESY spectra between hydroxy protons from sucrose and from lactose. Comparison of Figure 5c,d and Figure 6c,d shows that the cross-peaks are stronger with trehalose. The GlcOH2 and FruOH1' chemical exchange in sucrose has the strongest intensity. Weak intermolecular exchanges between GlcOH3 and OH4 of sucrose and GalOH3' and OH6' of lactose are found. The ROESY spectra of the lactose/trehalose/water and lactose/sucrose/water systems shows that it is the same hydroxy protons in lactose that are involved in chemical exchange with trehalose as well as sucrose.

## 3. Experimental

### 3.1. Sample Preparation

Trehalose, lactose, sucrose and glucose were of analytical grade, and used without further purification. For the diffusion studies the samples were prepared using 99.9% D_2_O (Cambridge Isotope Labs, Andover, MA, USA) and 500 μL of the samples were transferred to 5 mm NMR tubes. The following samples were prepared; lactose: 34 and 253 mg/mL; trehalose: 34, 258 and 342 mg/mL; trehalose/lactose: 34/34, 170/170, 254/254 and 780/63 mg/mL; trehalose/glucose: 35/20 and 785/67 mg/mL. The samples for studies of hydroxy protons were prepared using milli-Q water and 10% acetone-*d*_6_ and 600 μL of the solutions were transferred into 5 mm NMR tubes. In order to minimize absorption of impurities from glassware, the NMR tubes were soaked prior to use for a minimum of 1 h in a 100 mM solution of sodium phosphate buffer, pH 7.0, and then rinsed with milli-Q water [64]. The pH of the sample solutions was adjusted to an optimum value for observation of hydroxy protons (pH 6–7) by addition of aliquots of 10 mM sodium hydroxide or hydrochloric acid solutions. The concentration of the samples for the hydroxy proton studies were 35 and 203 mg/mL for lactose and 35, 206 and 338 mg/mL for trehalose. For the trehalose/lactose mixtures, the concentrations were 37/36, 256/256, 383/380 and 593/54 mg/mL of the respective disaccharide. The sucrose and glucose samples were prepared to a concentration of 202 mg/mL whereas the sucrose/lactose and glucose/lactose mixtures had concentrations of 178/181 and 239/240 mg/mL respectively. For the highest sugar concentrations, the samples were dissolved using heating, stirring and ultrasound bath.

### 3.2. NMR Experiments

The self-diffusion NMR experiments were performed on a Bruker DRX-400 MHz spectrometer using a 5.0 mm ^1^H/^13^C/^15^N/^31^P QNP probe equipped with a z-gradient and controlled by Topspin 1.3 software. The maximum gradient strength of the probe was 50 G/cm. The diffusion data was acquired using the ledbpgp2s [65] pulse sequence from the Bruker sequence library. The diffusion delay (Δ) and the gradient pulse length (δ) were set to 100 and 2.2 ms respectively. The data was processed with the DOSY and T1/T2 processing tools available in Topspin. 

The NMR spectra of hydroxy protons were recorded on a Bruker Ascend 600 MHz spectrometer using a 5 mm ^1^H/broadband “Smartprobe” equipped with z-gradient and controlled with Topspin 3.1. The Watergate [66] and excitation sculpting [67] pulse sequences were used for solvent suppression. The ^1^H-NMR spectra were referenced by setting the residual acetone-*d*_5_ signal to δ_H_ 2.204 ppm. NMR spectra were recorded between −10 °C and +10 °C at 5 ° intervals in order to calculate the temperature coefficients. The chemical shifts, ROEs and chemical exchange of hydroxy protons were measured at −5 °C, ^3^*J*_CH, OH_ were extracted at −10 °C. TOCSY and ROESY spectra were recorded with at least 2K data points in the F2 dimension and 512 increments in F1. A minimum of 2 scans were used and the recycle delay was set to 1.5 seconds. TOCSY spectra for determination of δ and dδ/dΤ were recorded with 20 ms mixing time. ROESY spectra used for chemical exchange studies were recorded with 20, 40, 60, 80 and 100 ms mixing times. ROESY and TOCSY spectra were processed with 4Kx1K data points using a π/4 sine squared bell function in the F2 dimension and π/2 in F1. 

## 4. Conclusions

The aim of this study was to investigate if and how trehalose and sucrose affect the hydration and hydrogen bonding interaction in lactose using NMR of hydroxy protons. The temperature coefficients of the hydroxy protons in lactose were not significantly affected by the presence of trehalose or sucrose, indicating that these disaccharides do not affect the hydrogen bond network in lactose, at least in the concentration range investigated here. The hydroxy protons in lactose experienced a small upfield shift upon addition of trehalose, but also of sucrose. The hydroxy protons in trehalose and in sucrose were affected in a similar manner. This general effect on the chemical shift might be attributed to reduced hydration due to slowed down water dynamics. Simulations have shown [24,27] that trehalose in solutions with proteins create a shell of water around the protein with reduced mobility. The chemical shifts of the GlcOH3(α/β) signals were slightly less affected by the addition of trehalose or sucrose, attributed to the spatial proximity to GalO5' which protect GlcOH3 from the surroundings.

Although the chemical shift changes are small our data are in good agreement with a recent study by Lupi *et al*. [22] who suggested, using polarized light scattering and molecular dynamics simulations, that it is the concentration of hydroxy groups which is the relevant parameter guiding both water-sugar and sugar-sugar interactions more than the type of sugar. The authors showed that on average, for dilute solutions, the same number of water molecules, per hydroxy group, was dynamically retarded independently of the sugar size. This number was found to be larger than the number of water molecules directly hydrogen bonded to the sugars, indicating a dynamical hydration shell. The authors also suggest a scaling law based on the number of hydroxy groups that makes the number of dynamically perturbed water molecules independent of sugar size. This independence between the amount of dynamically perturbed water and glucose and trehalose is referred to as an unspecific solvation effect. 2D ROESY experiments revealed chemical exchange between the hydroxy protons of lactose and those of trehalose, the intensity of the exchange being stronger upon increasing the sugar concentration. Molecular dynamics simulation [31] have shown that trehalose molecules interact in solution through the OH2, 3 and 4 groups and it is also through these hydroxy protons that trehalose is interacting with lactose, probably through water.

Our study as well as the one by Lupi *et al*. [22] was performed for sugar concentrations ≤ 50% (w/w). From the spin relaxation rates of deuterated trehalose and ^17^O-enriched water measured over a wide range of concentrations (0.025–1.47 M) Winther *et al*. [43] have recently reported that direct trehalose-trehalose interactions exist at both high and low sugar concentrations. These direct trehalose-trehalose interactions typically involved only a small part of the molecule, often just a single solute-solute hydrogen bond. At low concentrations, only a minor fraction of the trehalose molecules were involved in clusters. The hydration of trehalose was found to be unremarkable and similar to other organic solutes and no special hydration properties of the disaccharide could be identified. Lerbret *et al*. [14] have shown using low frequency VDOS and Raman scattering experiments that trehalose, maltose and sucrose induce a significant slowing down of water dynamics, which depends primarily on the total number of hydrogen bonds that they form with water. As a consequence, water diffusion significantly slows down when the sugar concentration increases, following an increase of water-water hydrogen bond lifetimes. This induced retardation of water dynamics is strongly amplified at concentrations above 40% (w/w) by the percolation of the hydrogen bond network of sugars [12]. This concentration corresponds approximately to the concentration threshold where slight differences between the influences of the respective sugars start to appear. Thus for higher concentration of sugars (>50% (w/w)) the effects of trehalose and sucrose on the hydration and hydrogen bonding interaction in lactose might well be differentiated, but this could not be investigated due to the low temperatures required for NMR studies of hydroxy protons.
